# MRTF-A promotes angiotensin II-induced inflammatory response and aortic dissection in mice

**DOI:** 10.1371/journal.pone.0229888

**Published:** 2020-03-24

**Authors:** Sohei Ito, Yohei Hashimoto, Ryohei Majima, Eichi Nakao, Hiroki Aoki, Michihide Nishihara, Satoko Ohno-Urabe, Aya Furusho, Saki Hirakata, Norifumi Nishida, Makiko Hayashi, Koichiro Kuwahara, Yoshihiro Fukumoto

**Affiliations:** 1 Division of Cardiovascular Medicine, Department of Internal Medicine, Kurume University School of Medicine, Kurume, Japan; 2 Cardiovascular Research Institute, Kurume University, Kurume, Japan; 3 Department of Cardiovascular Medicine, Shinshu University School of Medicine, Matsumoto, Japan; Brigham and Women's Hospital, Harvard Medical School, UNITED STATES

## Abstract

Aortic dissection (AD) is a major cause of acute aortic syndrome with high mortality due to the destruction of aortic walls. Although recent studies indicate the critical role of inflammation in the disease mechanism of AD, it is unclear how inflammatory response is initiated. Here, we demonstrate that myocardin-related transcription factor A (MRTF-A), a signal transducer of humoral and mechanical stress, plays an important role in pathogenesis of AD in a mouse model. A mouse model of AD was created by continuous infusion of angiotensin II (AngII) that induced MRTF-A expression and caused AD in 4 days. Systemic deletion of *Mrtfa* gene resulted in a marked suppression of AD development. Transcriptome and gene annotation enrichment analyses revealed that AngII infusion for 1 day caused pro-inflammatory and pro-apoptotic responses before AD development, which were suppressed by *Mrtfa* deletion. AngII infusion for 1 day induced pro-inflammatory response, as demonstrated by expressions of *Il6*, *Tnf*, and *Ccl2*, and apoptosis of aortic wall cells, as detected by TUNEL staining, in an MRTF-A-dependent manner. Pharmacological inhibition of MRTF-A by CCG-203971 during AngII infusion partially suppressed AD phenotype, indicating that acute suppression of MRTF-A is effective in preventing the aortic wall destruction. These results indicate that MRTF-A transduces the stress of AngII challenge to the pro-inflammatory and pro-apoptotic responses, ultimately leading to AD development. Intervening this pathway may represent a potential therapeutic strategy.

## Introduction

Aortic dissection (AD) is a serious aortic disease which often results in a lethal outcome [1]. Although establishing preventive and therapeutic strategies is an urgent issue for AD, it is hampered by the elusive molecular pathogenesis of AD. Several genetic disorders are known to predispose individuals to aortic aneurysm and AD [2]. The best characterized genetic disorders for familial AD are mutation of *FBN1* that causes Marfan syndrome, and mutations of *TGFB2*, *TGFBR1*, *TGFBR2* and *SMAD3* that cause Loeys-Dietz syndrome, underscoring the importance of TGF-β pathway for the homeostasis of aortic walls. Another group of culprit genes for familial AD are those encoding contractile proteins in smooth muscle cells (SMCs), including smooth muscle α-actin (*ACTA2*), myosin heavy chain (*MYH11*), and those encoding the regulators of SMC contraction, including myosin light chain (*MYLK*) and cyclic GMP-dependent protein kinase (*PRKG*), indicating the importance of SMC cytoskeleton in pathogenesis of AD. Currently it is unclear how abnormalities in SMC cytoskeletal proteins result in AD development.

Studies in human AD samples and animal AD models indicate that inflammatory response plays a central role in the pathogenesis of AD [3, 4]. We also reported the involvement of inflammatory response in mouse models [5–7] and in human AD tissue [8]. Although these studies demonstrated the importance of inflammation, it remains elusive how inflammatory response is initiated or whether and how it is related to SMC cytoskeleton.

Recently, myocardin-related transcription factor (MRTF) cofactors were proposed to transduce neurohumoral and mechanical stimuli to stress response [9]. Specifically, neurohumoral and mechanical stimuli to cells promote Rho family GTPases-dependent actin polymerization that liberates MRTF to enter the nuclei and activate serum response factor (SRF)-dependent gene expression[9]. A series of studies have shown that MRTF-A, a member of MRTF family, regulates cardiovascular inflammation and remodeling in response to mechanical and humoral stimuli [10–12]. Therefore, MRTF-A is an attractive candidate that links insults on aortic wall, actin cytoskeleton, and inflammatory response in the context of AD pathogenesis. Current study was designed to test if MRTF-A is involved in the molecular pathogenesis of AD, using angiotensin II-induced mouse model of AD [13] with genetic deletion [11] or pharmacological inhibition [14] of MRTF-A.

## Materials & methods

### Animal experiments

All animal experimental protocols were approved by the Animal Experiments Review Boards of Kurume University. Mice with genetic deletion of *Mrtfa* (MRTF-A-KO) was created as previously described [11] and backcrossed to C57BL6/J for more than 5 generations. All animal experiments ware done in male mice at the age of 10–11 weeks, as AD predominantly affects men [15]. C57BL/6J mice were purchased from Charles River Laboratories. We used wild type (WT) C57BL/6J mouse as a control for MRTF-A-KO mouse to facilitate the experiments and minimize the number of mice to sacrifice, because the reproductivity of MRTF-A-KO line was low in our hands. Angiotensin II (AngII, 1 μg/kg/min, Peptide Institute #4001, Osaka, Japan) was subcutaneously infused using osmotic minipumps (Durect Alzet #1007D, Cupertino, CA) for 4 days. Implantation of the minipumps filled with saline did not cause discernible aortopathy (S1 Fig). Mice were administered twice daily with 300 mg/kg CCG-203971 (Cayman Chemical #15075, Ann Arbor, MI) in 50 μL dimethylsulfoxide (DMSO) or 50 μL DMSO alone. Administration of CCG-203971 or DMSO was started 12 hours before starting AngII infusion and continued throughout the observational period. Systolic blood pressure was measured by the tail-cuff method (BP-98A, Softron, Tokyo, Japan).

At the end of experimental periods, mice were killed by pentobarbital overdose. For the histological analysis, we excised aortae 4 days after AngII infusion. For the expression analysis we obtained aortae with or without 1 day AngII infusion. The aortic samples were obtained after phosphate-buffered saline (PBS) perfusion at the physiological pressure from the root of aorta to just bellow subclavian artery branch, frozen in liquid nitrogen, and stored at -80˚C until analysis. We used 4% paraformaldehyde in PBS for histological analysis. For the expression analysis, perfusion was performed with PBS. For quantification of the tear area, 200 μl of 1% Evans blue solution was administered from orbital venous plexus before pentobarbital overdose, followed by PBS perfusion to wash out excessive Evans blue.

### Quantification of area of tear and hematoma

Excised aortae was opened, pinned and photographed under natural light and 488 nm excitation light to obtain the fluoresce of Evans blue. We measured the area of tear and hematoma using Image-Pro PLUS software version 6 (Media Cybernetics, Rockville, MD, USA). We also measure the area of ascending aorta defined as an area from the root of aorta to just bellow subclavian artery branch.

We defined aortic dissection (AD) as the lesion with the intramural hematoma connected to the intimal tear. When the intimal tear was visualized by Evans blue perfusion, intramural hematoma was always associated with the intimal tear. Our collaborators also reported that the intramural hematoma in this model was associated with the disruption of intima-medial elastic lamellae by propagation-based phase-contrast synchrotron imaging [16]. As for the incidence of AD, the presence of AD was determined by the presence of intramural hematoma in the macroscopic images.

### Expression analysis

For protein and mRNA expression analysis, aortic samples were pulverized using SK Mill (Tokken, Kashiwa, Japan), and the proteins were extracted with RIPA buffer. After resolving the proteins using the NuPAGE electrophoresis system (Invitrogen, Carlsbad, CA), western blotting was performed using antibodies to Myc (0.5 μg/mL, Cell Signaling Technology #9402, Danvers, MA), Stat3 (0.5 μg/mL, Cell Signaling Technology #12648), phospho-Stat3 (P-Tyr705, 0.5 μg/mL, Cell Signaling Technology #9145), MRTF-A (0.56 μg/mL, Proteintech #21166-1-AP, Rosemont, IL) and Gapdh (0.2 μg/mL, Merck Millipore #MAB374, Temecula, CA). As we noticed in preliminary study that protein expression levels were highly variable within a experimental group, we used 16 to 18 samples in a given group for western blotting. Images of whole membranes are shown in S2 Fig.

For mRNA expression analysis, we used RNeasy kit (Qiagen, Hilden, Germany) to isolate total RNA. We performed transcriptome analysis using the SurePrint G3 Mouse Gene Expression v2 8x60K Microarray Kit (Agilent, Santa Clara, CA). The full dataset of the transcriptome analysis is available at Gene Expression Omnibus (accession # GSE138484). We performed the gene annotation enrichment analysis for the selected genes using Database for Annotation, Visualization and Integrated Discovery (DAVID) [17] with the Gene Ontology terms set to GOTERM_BP_FAT, GOTERM_CC_FAT and GOTERM_MF_FAT. The full lists of the DAVID analysis and corresponding gene descriptions are available as S1 and S2 Tables. mRNA expressions of *Il6*, *Ccl2*, *Tnf* and *Actb* were measured by quantitative real time polymerase chain reaction (qRT-PCR) using commercially available probes (primer set ID MA104898, MA108953, MA163499, MA050368: Takara Bio, Shiga, Japan).

### Histological analysis

We observed 5 μm sections of paraffin-embedded ascending aortic tissue with elastic van Gieson (EVG) or hematoxylin and eosin (H&E) staining. The tissue sections were also stained for MRTF-A (3.3 μg/mL, NOVUS Biologicals #NBP1-1968, Centennial, CO) with TSA-plus system (Perkin Elmer, Waltham, MA), smooth muscle α-actin (SMA, 0.2 μg/mL, Sigma-Aldrich, St Louis, MO) with Dylight 549-labeled secondary antibody (1:100, antibody concentration undetermined, Jackson ImmunoResearch #115-505-166, West Grove, PA), Cd31 (1.67 μg/mL, Abcam #ab56299, Cambridge, UK) with Cy3 conjugated secondary antibody (15 μg/mL, Jackson ImmunoResearch #712-165-153), Il6 (1:300, antibody concentration undetermined, Abcam #ab6672) with Cy3 conjugated secondary antibody and Myc (1 μg/mL, Cell Signaling Technology #9402) with Cy3 conjugated secondary antibody. Frozen ascending aortic tissue sections were stained for Cd45 (0.5 μg/mL, Biolegend #103101, San Diego, CA) with Cy3 conjugated secondary antibody. Isotype control antibodies were used for rabbit IgG (Abcam #ab37415) and Rat IgG2b kappa (Abcam #ab18541) at the same concentrations as the corresponding antibodies. For terminal deoxynucleotidyl transferase dUTP nick end labeling (TUNEL) assay, we used *in situ* Apoptosis Detection Kit (Takara Bio #MK500) with protease K (Takara Bio #9034) for antigen retrieval. Nuclei were stained with 4’,6-diamidino-2-phenylindole mounting media (DAPI, Vector Laboratories #H-1500, Burlingame, CA).

### Statistical analysis

All data are expressed as medians and interquartile ranges using box plots. Statistical analysis was performed with Mann-Whitney test for the comparisons of 2 groups. Kruskal-Wallis test was performed for multiple groups. Post-test analysis was performed by Dunn’s multiple-comparison test. P < 0.05 was considered significant.

## Results

### Role of MRTF-A in AngII-induced AD

**Fig 1:**
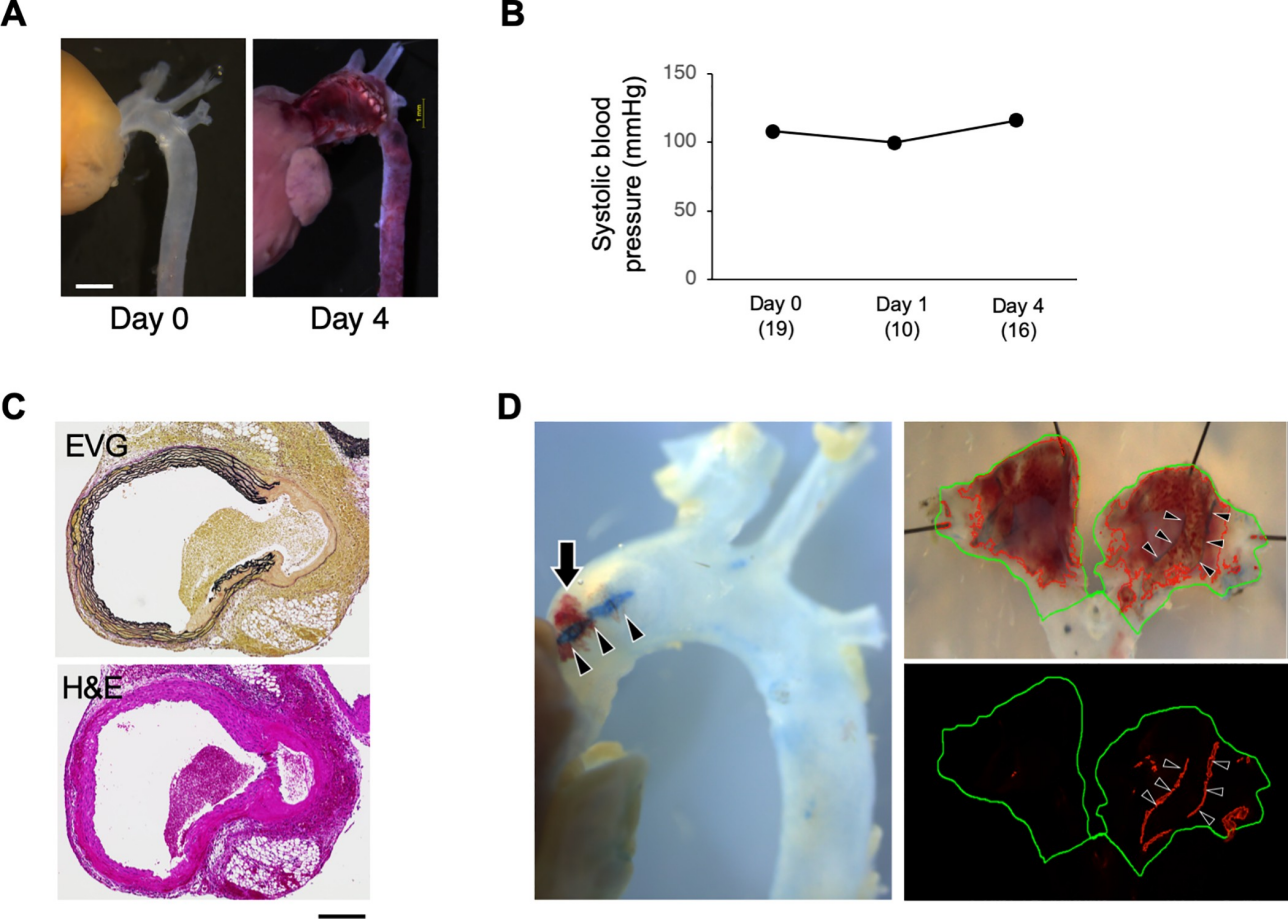
AD model by AngII administration. (A) Representative images are shown before (Control) and 4 days after starting AngII administration. Bar 1 mm. (B) Systolic blood pressure during the observational period. (C) Representative histological images with elastica van Gieson (EVG) and hematoxylin & eosin (H&E) staining 4 days after starting AngII administration. Bar 0.2 mm. (D) Evans blue-perfused aortae from WT mice 4 days after AngII infusion. Black arrow (left image): hematoma. Green line (upper and lower right images)s: area of ascending aorta. Red line (upper right image): area of hematoma. Arrowheads: Evans blue-stained tears in bright field images and red fluorescence in a dark field image.

We administered AngII (1 μg/kg/min) to WT mice that caused ascending aortic dissection (AD) in 4 days after AngII infusion (Fig 1A) without significant changes in systolic blood pressure (Fig 1B), as previously reported [18]. Our collaborators have demonstrated that this AD model is characterized by the progressive disruption of intima-medial elastic lamellae that is associated with the intramural hematoma by three dimensional imaging [16]. Consistently, our histological observation showed intramural hematomas and tears defined as disruption of elastic lamella at 4 days of AngII infusion (Fig 1C). For the quantitative analysis of AD, we measured the areas of intramural hematoma and intimal tear in the ascending aorta as visualized by Evans blue perfusion (Fig 1D).

**Fig 2:**
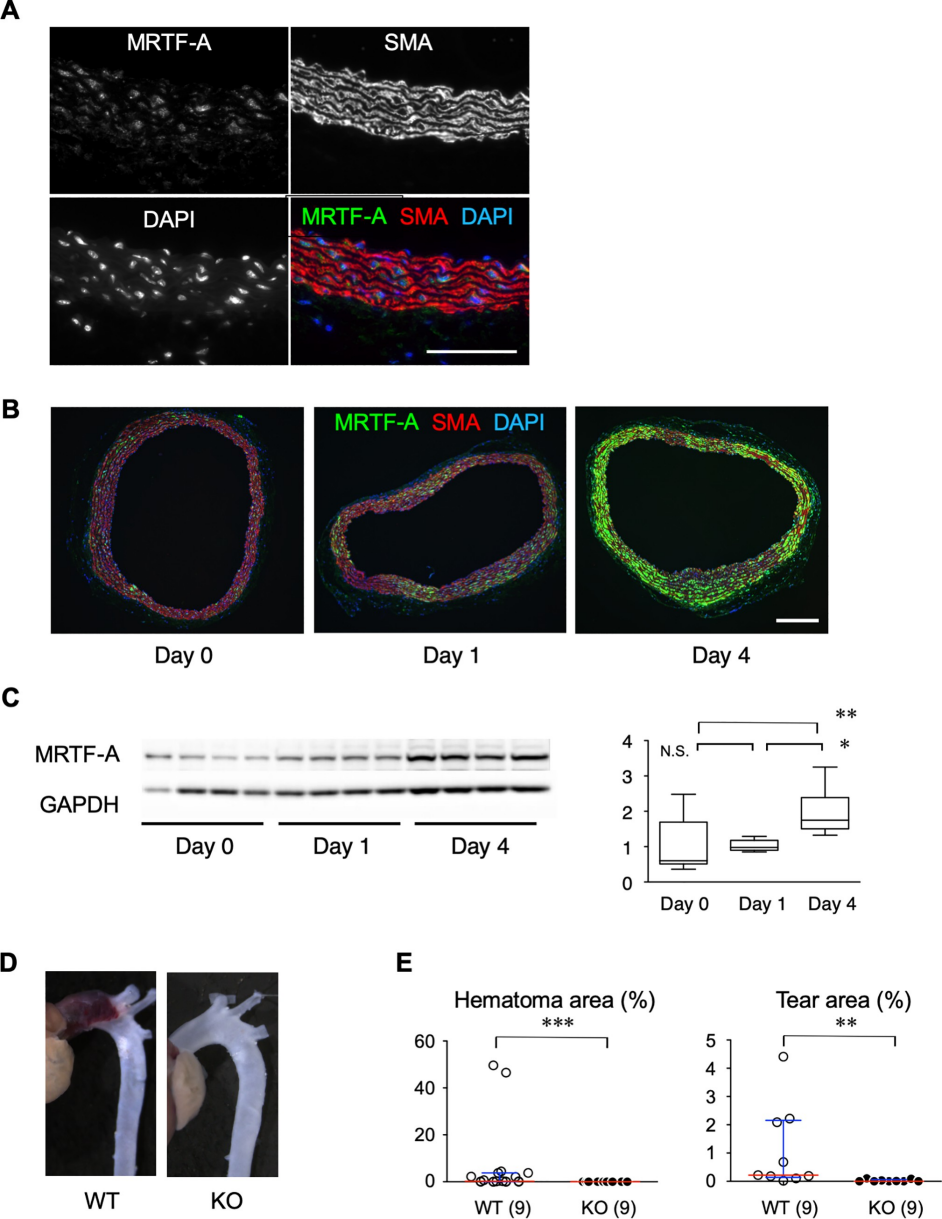
Protein expression and role of MRTF-A in AD model. (A) Ascending aortic sections were stained for MRTF-A, SMA and DAPI in WT mice 4 days after starting AngII administration. Bar 0.1 mm. (B) Immunofluorescence staining for MRTF-A and SMA with nuclear DAPI staining before, 1 day and 4 days after starting AngII infusion in WT ascending aorta. Bar 0.1 mm.(C) Immunoblot analysis for MRTF-A in the aortic tissue before, 1 day and 4 days after starting AngII infusion in WT ascending aorta. Representative immunoblot images and quantitative analysis are shown. Gapdh served as an internal loading control. (D) Representative images of aortas from WT and MRTF-A-KO mice AngII administered for 4 days. (E) Area ratio of hematomas and tears in the ascending aorta from WT or MRTF-A-KO mice. Red and blue bars indicate the medians and interquartile ranges, respectively. The numbers in parenthesis indicate the number of biological replicates. * P < 0.05, ** P < 0.01, *** P < 0.001.

To evaluate the localization of MRTF-A in mouse aorta, we performed immunofluorescence staining for MRTF-A and smooth muscle α-actin (SMA), a marker of smooth muscle cells (Fig 2A). MRTF-A staining was detected mainly in the medial layer and overlapped with part of SMA-positive smooth muscle cells in the aortic wall. AngII infusion induced MRTF-A protein expression mainly in the medial layer as demonstrated by immunofluorescence staining (Fig 2B). AngII-induced increase in MRTF-A expression was confirmed by western blotting (Fig 2C). To clarify the role of MRTF-A in AD model, we compared the AD phenotype in WT mice and MRTF-A knockout (MRTF-A-KO) mice (Fig 2D, Table 1). The incidence of AD in MRTF-A-KO mice (0.0%, n = 9) was significantly lower compared to WT mice (55.6%, n = 18; p<0.05). Quantitative analysis of hematoma and tear areas also demonstrated the significant suppression of AD phenotype in MRTF-A-KO mice (Fig 2E).

**Table 1:** Incidence of AD in mouse model.

Genotype Intervention	WT AngII + DMSO	KO AngII + DMSO	WT AngII + CCG
Total (n)	18	9	8
AD (n)	10	0	2
AD (%)	55.6	0.0 **	25.0

The incidence of AD is shown for each experimental group.

** P < 0.01 compared with WT (AngII + DMSO) by Fisher's exact test.

### Role of MRTF-A in gene expression during AD development

**Fig 3:**
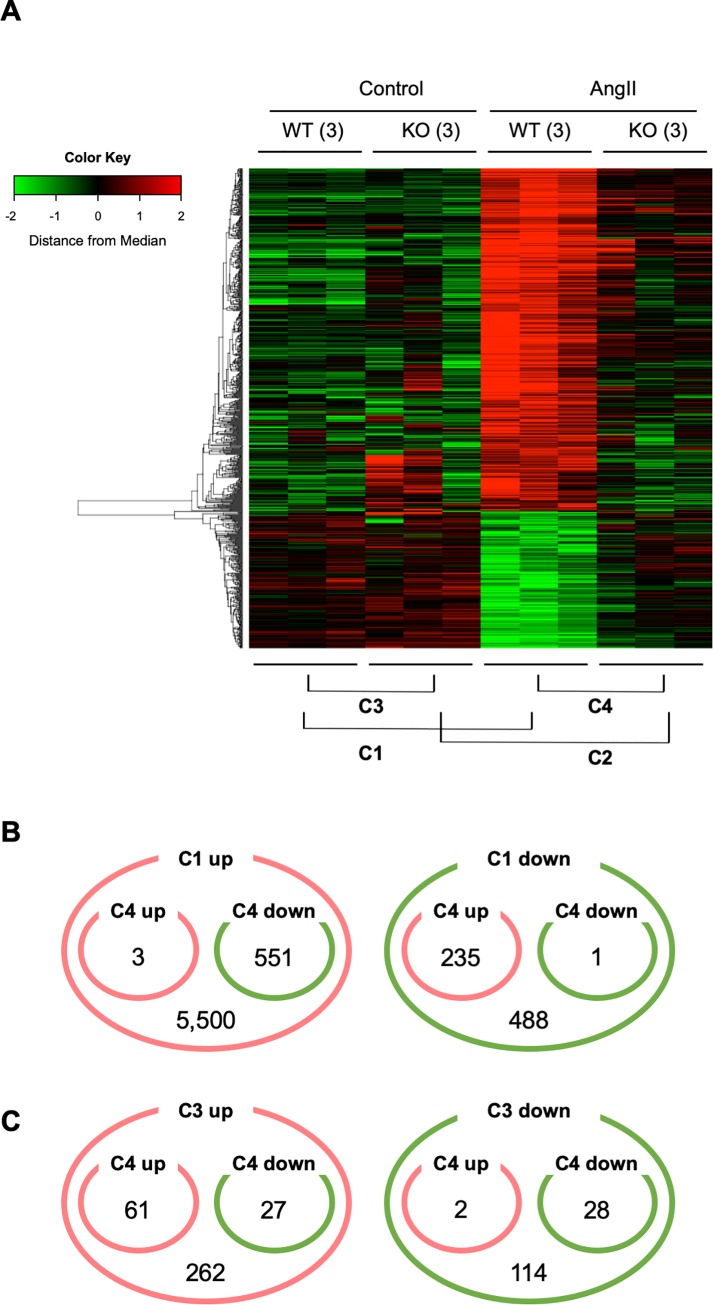
Transcriptome analysis of ascending aorta. (A) The result of hierarchical clustering analysis is shown for AngII- and MRTF-A-regulated genes by the heat map. The samples are from WT or MRTF-A-KO mice with or without AngII infusion for 1 day. The numbers in parenthesis indicate the number of biological replicates. Each gene is color-coded by red and green colors for the induction and the suppression, respectively, from the median. Comparison 1 (C1); without (Control) and with AngII in WT, Comparison 2 (C2); Control and AngII in MRTF-A-KO, Comparison 3 (C3); WT and MRTF-A-KO in Control, Comparison 4 (C4); WT and MRTF-A-KO in AngII. Comparison 2 was not used for further analysis. (B, C) Diagrams indicate number of genes with significant expression changes in each comparison as indicated in panel A. The numbers of upregulated (up) and downregulated (down) genes are shown.

To clarify the role of MRTF-A in AngII-induced aortic pathology, we performed transcriptome analysis of aortic tissues from WT and MRTF-A-KO mice with and without AngII. We performed the analysis at the first day of AngII administration before AD development to avoid the consequence of the catastrophic aortic wall destruction due to AD. We defined AD-related genes as those with significant changes by AngII (P < 0.05, fold change > 2 or < 0.5). Among the AD-related genes, we further defined MRTF-A-regulate genes, as those with significant changes between WT and MRTF-A-KO (Fig 3A). Within the AngII-sensitive genes, approximately 6,000 genes were upregulated and 700 genes were downregulated (Fig 3B). The effect of *Mrtfa* deletion was a partial suppression of the AngII-induced changes. In the absence of AngII challenge, approximately 350 genes were upregulated and 250 genes were downregulated by *Mrtfa* deletion (Fig 3C). These findings suggested that MRTF-A mediated part of AngII-induced changes in gene expression. We performed the gene annotation enrichment analysis for the selected genes using Database for Annotation, Visualization and Integrated Discovery (DAVID) [17]. The DAVID analysis revealed that AngII-induced, MRTF-A-dependent genes were highly enriched for inflammatory response, angiogenesis and cell death (Table 2). AngII-suppressed, MRTF-dependent genes were enriched for myofibril and muscle organ development (Table 3), although the enrichment scores were not as high as the AngII-induced genes.

**Table 2:** Down-regulated annotation clusters in MRTF-A-KO.

**Annotation Cluster 1 (18.28)**	**Annotation Cluster 10 (6.33)**
inflammatory response	blood vessel morphogenesis
defense response	Angiogenesis
immune response	vasculature development
**Annotation Cluster 2 (16.15)**	**Annotation Cluster 11 (5.73)**
response to cytokine	leukocyte chemotaxis
cellular response to cytokine stimulus	positive regulation of leukocyte migration
cytokine-mediated signaling pathway	chemokine-mediated signaling pathway
**Annotation Cluster 3 (11.13)**	**Annotation Cluster 12 (5.44)**
leukocyte migration	cytokine biosynthetic process
cell chemotaxis	cytokine metabolic process
leukocyte chemotaxis	regulation of cytokine biosynthetic process
**Annotation Cluster 4 (10.82)**	**Annotation Cluster 13 (5.43)**
defense response	cell activation
response to external stimulus	leukocyte activation
response to biotic stimulus	Hemopoiesis
**Annotation Cluster 5 (9.24)**	**Annotation Cluster 14 (5.37)**
regulation of cell proliferation	apoptotic process
cell proliferation	cell death
positive regulation of cell proliferation	programmed cell death
**Annotation Cluster 6 (7.46)**	**Annotation Cluster 15 (5.30)**
immune effector process	positive regulation of tumor necrosis factor superfamily cytokine production
regulation of immune response	positive regulation of tumor necrosis factor production
leukocyte mediated immunity	regulation of tumor necrosis factor superfamily cytokine production
**Annotation Cluster 7 (7.16)**	**Annotation Cluster 16 (5.20)**
regulation of chemokine production	regulation of cell communication
chemokine production	regulation of signaling
positive regulation of chemokine production	regulation of signal transduction
**Annotation Cluster 8 (7.06)**	**Annotation Cluster 17 (5.12)**
cytokine production	myeloid leukocyte activation
regulation of cytokine production	regulation of leukocyte mediated immunity
positive regulation of cytokine production	regulation of myeloid leukocyte mediated immunity
**Annotation Cluster 9 (6.69)**	
Hemopoiesis	
leukocyte differentiation	
immune system development	

Gene annotation enrichment analysis was performed for the genes with lower expression in MRTF-A-KO aorta compared to wild-type (WT) aorta, among the AngII-induced genes.

**Table 3:** Up-regulated annotation clusters in MRTF-A-KO.

**Annotation Cluster 1 (2.72)**
contractile fiber part
Myofibril
contractile fiber
**Annotation Cluster 2 (2.21)**
muscle organ development
skeletal muscle tissue development
skeletal muscle organ development
**Annotation Cluster 3 (2.08)**
alkali metal ion binding
T-tubule
Sarcolemma

Gene annotation enrichment analysis was performed for the genes with higher expression in MRTF-A-KO aorta compared to wild-type (WT) aorta, among the AngII-suppressed genes.

### Role of MRTF-A in aortic wall inflammation and apoptosis

**Fig 4:**
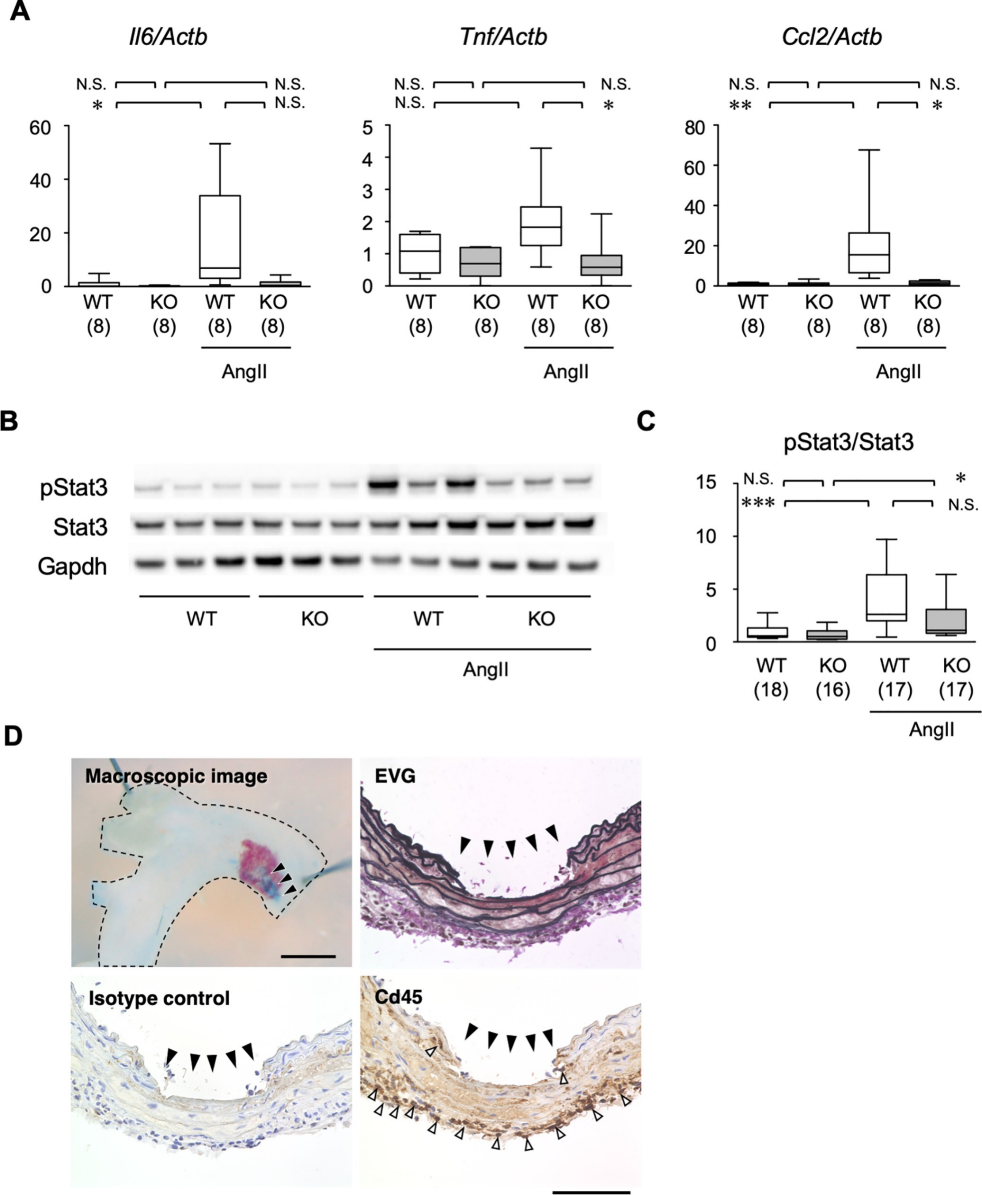
Inflammatory response in mouse AD model. (A) mRNA expressions were examined by qRT-PCR for *Il6*, *Tnf* and *Ccl2*, and normalized by *Actb*. The samples were obtained from WT or MRTF-A-KO mice with or without AngII administration for 1 day. (B, C) Protein expressions were examined by western blotting for pStat3, Stat3 and Gapdh. Representative images (B) and quantitative analysis (C) of western blotting are shown. The samples were ascending aortae from WT or MRTF-A-KO mice with or without AngII administration for 1 day. The numbers in parenthesis indicate the number of biological replicates. * P < 0.05, ** P < 0.01, *** P < 0.001, N.S. not significant. (D) Macroscopic and histological images are shown with EVG staining and Cd45 immunohistochemical staining along with the isotype control. Dotted line in the macroscopic image demarcates the aortic tissue. Aortic samples were obtained at day 4 of AngII infusion. Black and white arrowheads indicate the intimal tear and Cd45-positive staining, respectively. Bar 0.1 mm.

As the transcriptome analysis suggested that MRTF-A regulates the inflammatory response that plays a central role in AD pathogenesis [3, 4], we performed quantitative analysis of inflammatory cytokines by qRT-PCR in aortic tissue (Fig 4). The mRNA expressions of *Il6* and *Ccl2* were induced by 1 day of AngII infusion in WT aorta, while this response was not observed in MRTF-A-KO mice (Fig 4A). AngII challenge did not significantly alter *Tnf* expression, although MRTF-A-KO aorta showed lower *Tnf* expression in the presence of AngII. AngII-induced and MRTF-A-dependent inflammatory response was also demonstrated at the protein level by the western blotting for activated (phosphorylated) Stat3 (Fig 4B and 4C). At the tissue level, infiltration of CD45-positive inflammatory cells was demonstrated near the site of intimal tear at day 4 of AngII infusion (Fig 4D).

**Fig 5:**
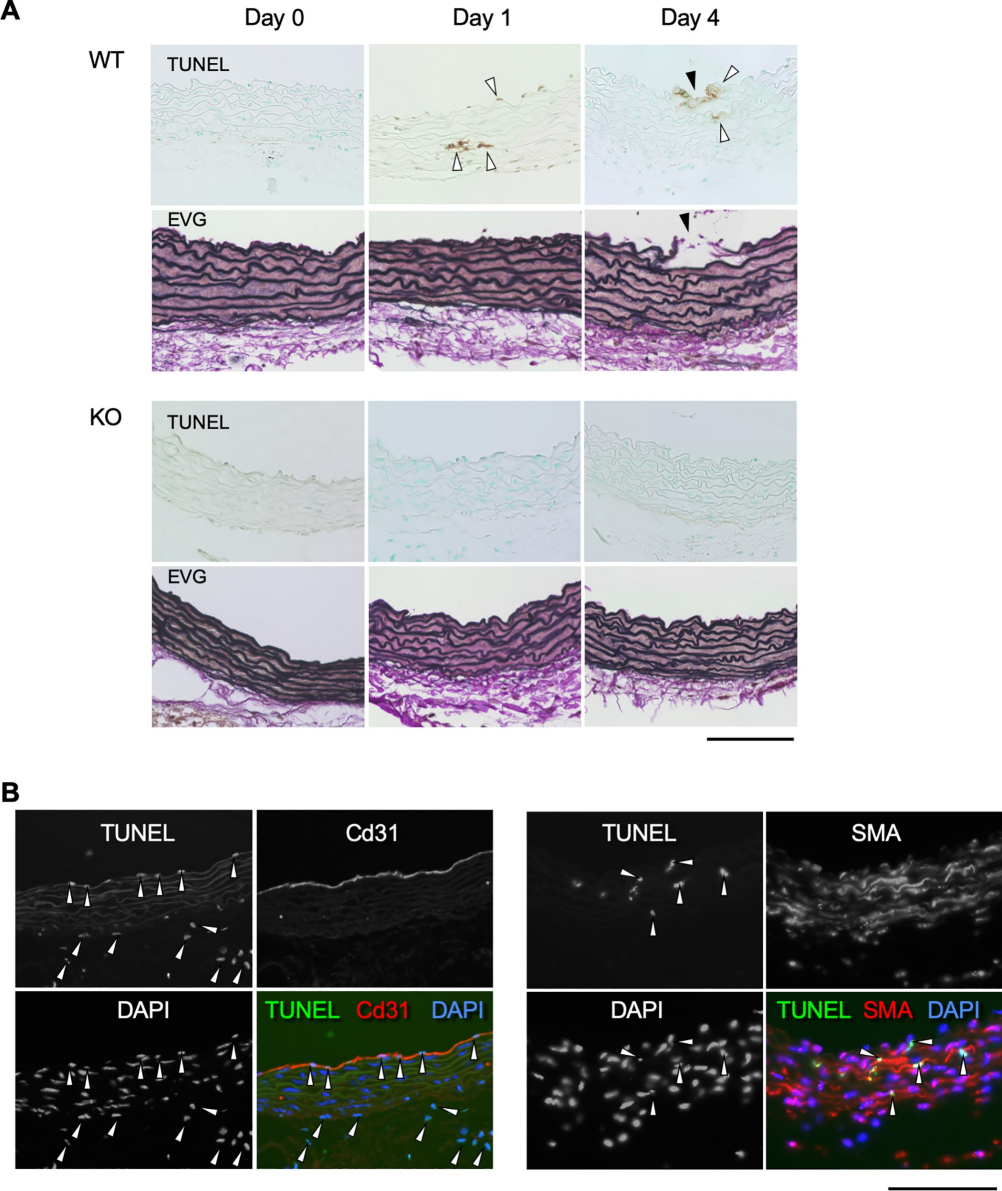
Apoptosis in mouse AD model. (A) Representative histological images are shown with TUNEL and EVG staining. The samples are from WT or MRTF-A-KO mice before (Control) and 1–4 days after starting AngII administration. White arrowheads indicate TUNEL-positive cells. Black arrowheads indicate the tear. (B) Immunofluorescence staining for Cd31, SMA, TUNEL and DAPI on ascending aortae are shown from WT mice 1 day after AngII administration. White arrowheads indicate TUNEL-positive nuclei. Bars 0.1 mm.

We next focused on apoptosis that may participate in AD pathogenesis [19]. We performed TUNEL staining to detect the double strand break of DNA, a marker of apoptotic cells, in WT or MRTF-A-KO aorta before, 1 day and 4 days after AngII infusion (Fig 5A). TUNEL-positive cells were not observed in either WT or MRTF-A-KO aortic tissue before AngII infusion. WT aortic tissue exhibited TUNEL-positive cells in the intimal and medial layers at day 1, and at the site of intimal tear at day 4 of AngII infusion, whereas MRTF-A-KO aorta did not. Double fluorescence labeling with cell type markers revealed the TUNEL staining colocalized with endothelial cell marker Cd31 and smooth muscle cell marker SMA (Fig 5B).

**Fig 6:**
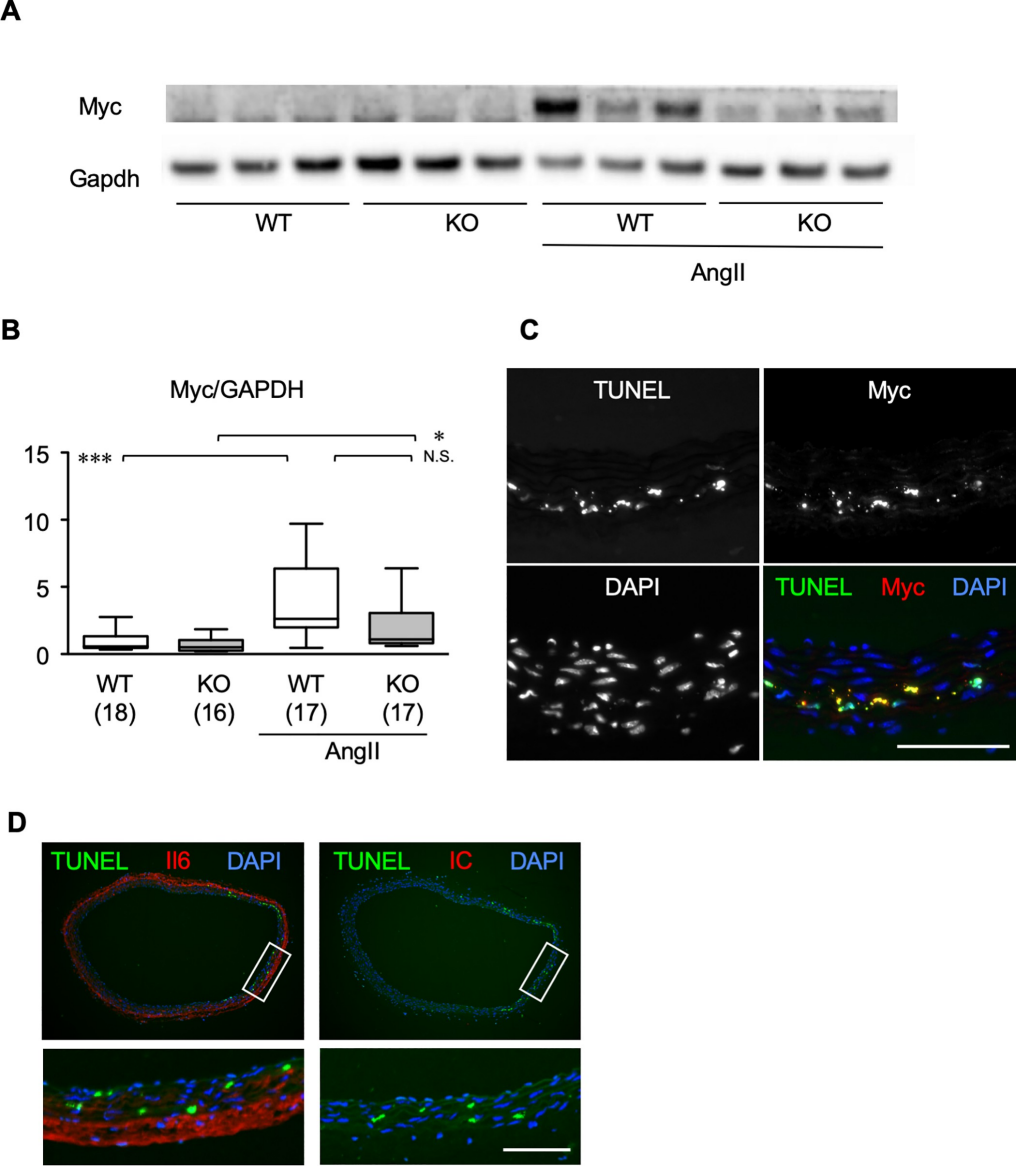
Myc and apoptosis in mouse AD tissue. (A, B) Protein expressions are shown by western blotting for Myc and Gapdh. Representative images (A) and quantitative analysis (B) are shown using ascending aortae from WT or MRTF-A-KO mice with or without AngII administration for 1 day. The numbers in parenthesis indicate the number of biological replicates. * P < 0.05, *** P < 0.001 compared with WT control. (C) Fluorescence staining for Myc, TUNEL, and DAPI on ascending aorta are shown from WT mice 1 day after AngII administration. (D) Fluorescence stainings are shown for Il6 and TUNEL in mouse aorta 1 day after starting AngII administration. Staining with rabbit IgG isotype control (IC) is also shown. White rectangles correspond to the magnified images at the bottom of the panels. Bar 0.1 mm.

To better characterize the MRTF-A-dependent inflammation and apoptosis, we examined the protein expression of Myc, a well-characterized regulator of apoptosis [20], as *Myc* mRNA expression was induced by AngII in an MRTF-A-dependent manner in our transcriptome analysis. Western blotting revealed that Myc protein was induced by AngII infusion in WT aorta, and to the lesser extent in MRTF-A-KO aorta (Fig 6A and 6B), suggesting that Myc induction by AngII was partly mediated by MRTF-A. Double fluorescence labeling revealed the colocalization of TUNEL staining and Myc expression (Fig 6C), consistent with the notion that Myc regulates apoptosis pathway [20]. Tissue localization of TUNEL positive cells in the aortic medial layer was closely correlated with the Il6 expression in the adventitial layer (Fig 6D).

### Pharmacological MRTF-A inhibition in AD

**Fig 7:**
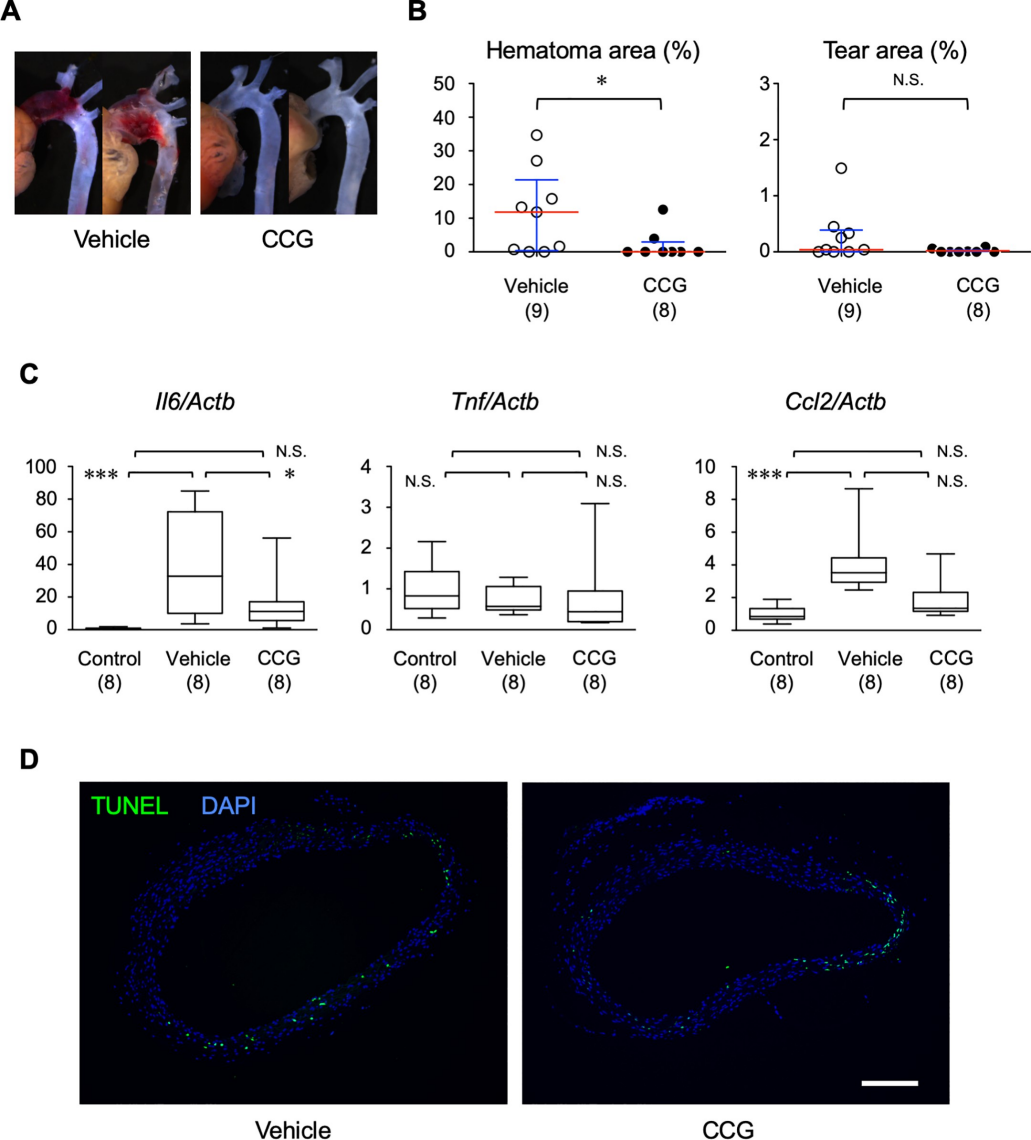
Pharmacological inhibitor of MRTF-A suppressed aortic dissection. Hematomas and tears in the ascending aorta from WT mice with vehicle (DMSO) or CCG-203971 treatment 4 days after starting AngII infusion. Representative images (A) and quantitative analysis for the hematoma and the tear (B) are shown. Bar 1 mm. Red and blue bars indicate the medians and interquartile ranges, respectively. (C) mRNA expressions were examined by qRT-PCR for *Il6*, *Tnf* and *Ccl2*, and normalized by *Actb*. The samples were obtained from WT with AngII administration for 1 day with or without CCG-203971 treatment. The numbers in parenthesis indicate the number of biological replicates. * P < 0.05, *** P < 0.001. N.S. not significant. (D) TUNEL and DAPI staining of aortic samples with AngII administration for 1 day with or without CCG-203971 treatment. Bar 0.1 mm.

The results thus far indicated that MRTF-A promotes AD development. Recently, CCG-203971, a specific inhibitor of MRTF-A-dependent transcription [14], has been reported to prevent the adverse tissue remodeling during inflammatory conditions [21]. To test the effect of the acute MRTF-A inhibition and to explore the clinical implication of our findings, we examined the effect of pharmacological MRTF-A inhibition on the AD model by treating WT mice with CCG-203971. The difference in the prevalence of AD between CCG-203971-treated group and vehicle-treated group did not reach statistical significance (Fig 7A, Table 1). CCG-203971 significantly suppressed the area of intramural hematoma by AD, whereas its effect on the tear area did not reach statistical significance (Fig 7B). AngII infusion caused induction of *Il6* and *Ccl2*, which was not observed in mice treated with CCG-203971 (Fig 7C). On the other hand, CCG-203971 did not significantly alter the appearance of TUNEL-positive cells at day 1 of AngII infusion (Fig 7D). These results indicated that pharmacological inhibition of MRTF-A by CCG-203971 partially suppressed AD phenotype.

## Discussion

The main finding in this study was that MRTF-A mediates inflammatory and apoptotic responses, and promotes AD development by AngII challenge. The inflammatory and apoptotic responses occurred at day 1, as indicated by the gene expression and histological analyses. Because the appearance of aortic wall tear, as detected by the Evans blue perfusion, occurred at day 4 of AngII infusion, inflammatory and apoptotic responses likely precede AD development. The involvement of MRTF-A in AD pathogenesis was further supported by the fact that CCG-203971, an inhibitor of MRTF-A, prevented the AngII-induced AD development. As recent studies indicated the involvement of inflammatory response [5, 6, 22, 23] and apoptosis [19, 24] in AD pathogenesis, these data suggested that MRTF-A promotes AD by regulating inflammatory and apoptotic responses.

Proinflammatory molecules, including secreted cytokines and chemokines, and intracellular signaling molecular including Stat3 and NFκB play critical roles in AD pathogenesis [4–6, 23, 25].[19] These molecules are activated by, and regulate the expression of cytokines such as Il6 and chemokines such as Ccl2 to orchestrate the behavior of resident cells including smooth muscle cells and infiltrating inflammatory cells [5, 23, 26]. Consistent with this notion, we found MRTF-A regulate expression of *Il6* and *Ccl2*, and infiltration of Cd45-positive inflammatory cells in the AD model. On the other hand, a previous report described that Cd45-positive cells appeared only sparsely at the late stage of AngII-induced aortopathy [18]. This apparent discrepancy may be explained by the fact that we observed specifically at the site of intimal tear whereas the previous report seems to show the aortic tissue without apparent tear. These findings suggest that spacio-temporal analysis of cellular dynamics would be required to understand the molecular pathogenesis of AD.

Apoptosis of aortic cells, mainly SMCs, is observed both in human AD and mouse model of AD [24], and tightly coupled with the phenotype of AD in mouse model [19, 27, 28]. Causative involvement of apoptosis in aortopathy was demonstrate by the suppression of SMC apoptosis and thoracic aortic aneurysm by a caspase inhibitor in a mouse model of Marfan syndrome [29]. These findings support the notion that pro-inflammatory and pro-apoptotic responses are the central biological process in pathogenesis of AD. Consistent with this notion, we observed TUNEL-positive apoptotic cells in aortic media appeared at the same time with adventitial expression of Il6. On the other hand, while the localizations of apoptotic cells and Il6 expression were close to each other, they did not overlap. Therefore, a molecular and cellular network is likely to underlie the manifestation of apoptosis and inflammation as a stress response in AD pathogenesis [19]. The findings we obtained by MRTF-A inhibition suggest that pro-inflammatory and pro-apoptotic function is the basis of the MRTF-A-mediated AD development.

The role of MRTF-A in adverse cardiovascular remodeling is consistent with previous reports [10–12, 30]. Mechanistically, MRTF-A is regulated by dynamics of actin cytoskeleton [31] to transduce neurohumoral and mechanical stimuli to the stress response [12]. In this regard, it is noteworthy that genetic abnormalities of SMC cytoskeletal and contractile proteins predispose the patients to AD [32]. Although it is unclear how mutations in cytoskeletal and contractile protein genes lead to AD development, current results suggest that abnormality in actin-dependent regulation of MRTF-A might be one of the mechanisms for the AD susceptibility.

The biological process under the control of MRTF-A seems context-dependent. MRTF-A is reported to mediate pro-inflammatory signal in various cell types including vascular SMCs [10], macrophages [33], and glomerular mesangial cells [34]. In the context of diseases, MRTF-A is pro-inflammatory in atherosclerosis [11, 35], liver injury [36], and inflammatory bowel disease [37]. On the other hand, MRTF-A exerts anti-inflammatory effect in pulmonary arterial SMCs [38]. MRTF-A is neuroprotective in ischemic cerebral injury possibly by inhibiting neuronal apoptosis [39, 40]. MRTF-A also prevents myocardial cell apoptosis [41]. Within the context of anticancer drug doxorubicin effect, MRTF-A can either cell-protective [42] or cell-toxic [43]. Of note, MRTF-A promotes atherogenesis by promoting macrophage survival and proliferation, and inflammatory response [35], exemplifying the complex role of MRTF-A and the relationship between inflammation and apoptosis.

Our data demonstrated that acute pharmacological inhibition of MRTF-A was effective in preventing AD. This finding suggests that MRTF-A plays an acute role in AD development, probably by promoting inflammation and apoptosis as discussed above. While MRTF-A protein was induced by AngII at day 4, AngII-induced *Il6* and *Ccl2* expressions were suppressed at day 1 by *Mrtfa* deletion or CCG-203971. Therefore, AngII is likely to activate MRTF-A both at the expression and functional levels. Whether MRTF-A is the trigger of AD initiation or the promoter of AD development awaits further study to delineate the chronological and functional sequence of the molecular events during AD development. Although our findings suggest that MRTF-A inhibition is a potential therapeutic opportunity for AD, the AD-inhibiting effect of CCG-203971 was not as complete as *Mrtfa* deletion. This may be due to the insufficient inhibition of MRTF-A by CCG-203971 during AD development. Alternatively, whole body *Mrtfa* deletion may developmentally alter the function of cells and tissue [44–46], which may affect AD pathogenesis. In addition, prevention of AD in the clinical situation is highly challenging. The annual incidence of AD in the general population is only 6 in 100,000, and prediction of AD is currently difficult, if not impossible [47]. Those who with genetic predisposition to AD, including Marfan syndrome, Loeys-Dietz syndrome and other forms of familial thoracic aortic aneurysm/dissection, may benefit from MRTF-A inhibition for preventing the development of life-threatening AD. However, even for the high risk population of AD, care should be taken for prophylactic chronic MRTF-A inhibition, because the function and its (patho)physiological role of MRTF-A seem context-dependent. Indeed, MRTF-A may play a protective role in neuronal and myocardial ischemia [30, 39, 40, 48, 49]. Another potential opportunity is the acute inhibition of MRTF-A after AD development to prevent further tissue destruction. For the MRTF-A inhibition therapy to realize, the role of MRTF-A needs to be clarified in the context of complex molecular and pathological changes during tissue destruction after AD onset.

In conclusion, our data demonstrated that MRTF-A promoted AD development possibly by activating proinflammatory and proapoptotic pathway. Further study for clarifying the cell type- and disease context-dependent function of MRTF-A would be essential for understanding the pathogenesis of AD, and for the development of preventive, diagnostic and therapeutic strategies for this fatal disease.

## Supporting information

S1 Fig Aortic morphology of mice implanted with saline-filled osmotic minipumps. No obvious aortopathy was observed. Bar 1 mm.
(TIFF)

S2 Fig Images of western blotting. Images of whole membranes: Membranes are shown for western blotting in Figs 2C, 4B and 6A. Red rectangles indicate the area that were used in corresponding figures. MW; molecular weight marker, LC; loading control. Detection of Myc band: Myc antibody revealed multiple bands in western blotting. True band was determined as the main band in lysate of proliferating smooth muscle cells. Bands with identical molecular weight were also observed in aortic tissue lysate with AngII stimulation, but not in that without AngII. Because *Myc* was induced by AngII, we concluded that the AngII-dependent bands was a true band of Myc, as indicated by arrowheads.
(TIFF)

S1 Table Gene annotation enrichment analysis. The table contains Entrez Gene IDs in the comparison groups shown in Fig 3B and 3C, and the results of the gene annotation enrichment analysis.
(XLSX)

S2 Table Genes in annotation clusters. The table contains Entrez Gene IDs and descriptions of genes in the annotation clusters obtained by the gene annotation enrichment analysis of the comparison groups shown in Fig 3B and 3C.
(XLSX)
