# Supplementary figures and images for: MRTF-A promotes angiotensin II-induced inflammatory response and aortic dissection in mice
Source: PLoS One. 2020 Mar 24;15(3):e0229888. doi: 10.1371/journal.pone.0229888 (PMC7092993; doi:10.1371/journal.pone.0229888)

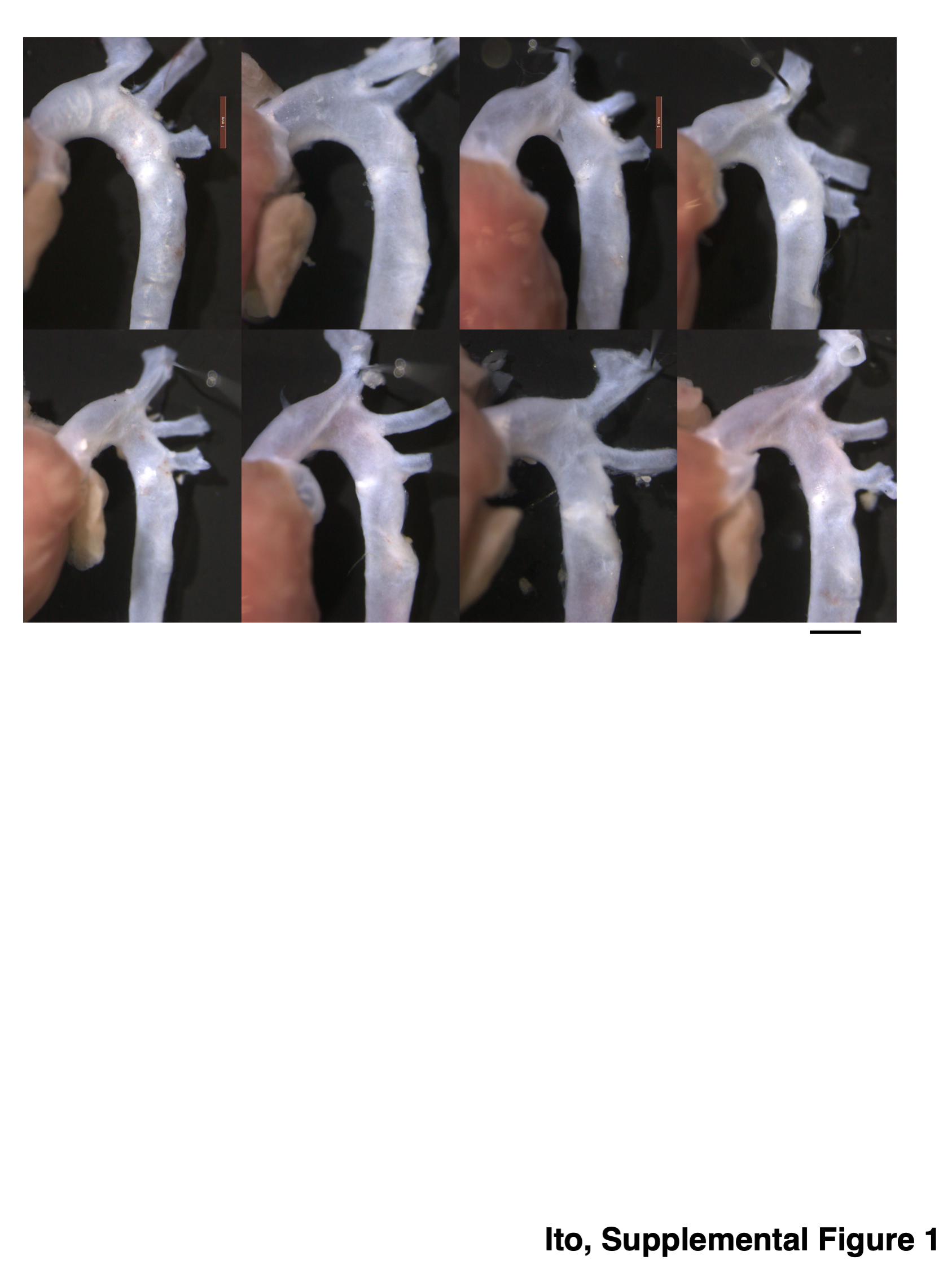

Supplement: S1 Fig — No obvious aortopathy was observed. Bar 1 mm. (TIFF) [file pone.0229888.s001.tiff]

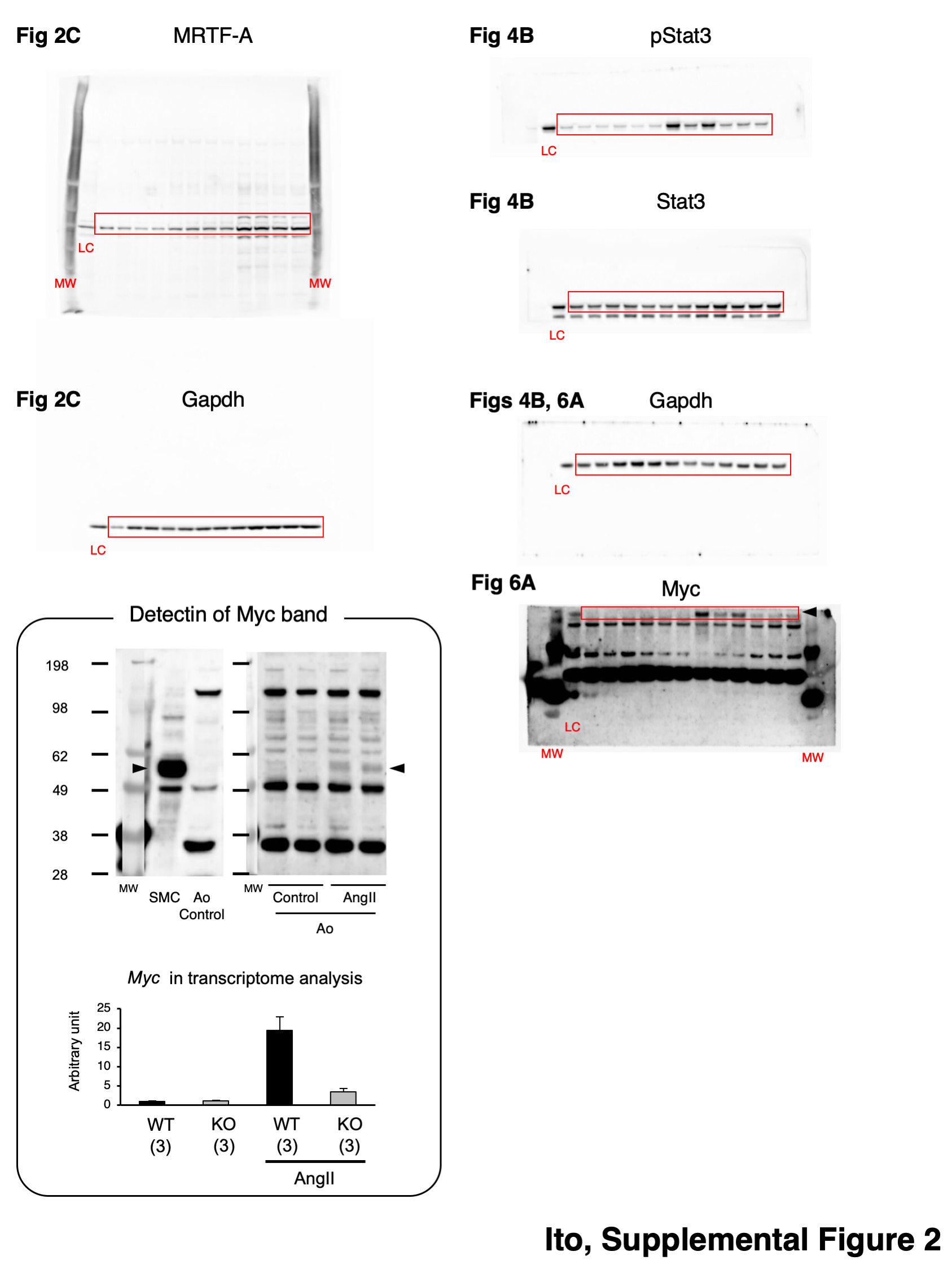

Supplement: S2 Fig — Images of whole membranes: Membranes are shown for western blotting in Figs 2C, 4B and 6A. Red rectangles indicate the area that were used in corresponding figures. MW; molecular weight marker, LC; loading control. Detection of Myc band: Myc antibody revealed multiple bands in western blotting. True band was determined as the main band in lysate of proliferating smooth muscle cells. Bands with identical molecular weight were also observed in aortic tissue lysate with AngII stimulation, but not in that without AngII. Because Myc was induced by AngII, we concluded that the AngII-dependent bands was a true band of Myc, as indicated by arrowheads. (TIFF) [file pone.0229888.s002.tiff]
